# Differences of Excess and Deficiency Zheng in Patients with Chronic Hepatitis B by Urinary Metabonomics

**DOI:** 10.1155/2013/738245

**Published:** 2013-04-24

**Authors:** Shujun Sun, Jianye Dai, Junwei Fang, Xiaojun Gou, Huijuan Cao, Ningning Zheng, Yang Wang, Wei Zhang, Yongyu Zhang, Wei Jia, Yiyang Hu

**Affiliations:** ^1^Center for Traditional Chinese Medicine and Systems Biology, Shanghai University of Traditional Chinese Medicine, Shanghai 201203, China; ^2^Institute of Liver Diseases, Shuguang Hospital, Key Laboratory of Liver and Kidney Diseases of Ministry of Education, Shanghai University of Traditional Chinese Medicine, Shanghai 201203, China; ^3^Liver Department, Longhua Hospital, Shanghai University of Traditional Chinese Medicine, Shanghai 201203, China; ^4^Department of Nutrition, University of North Carolina at Greensboro, North Carolina Research Campus, Kannapolis, NC 28081, USA

## Abstract

Traditional Chinese medicine (TCM) physicians stratify patients with the same disease into different subtypes in order to guide the appropriate treatment, which is called Zheng (TCM syndrome) classification. Excess and deficiency ZHENG is a couple of basic ZHENGs of maladjusted body nature, reflecting the struggling state of human body and pathogenic factor and is important and prevalently exists in the ZHENG classification of many diseases. The present work using chronic hepatitis B (CHB) as an entry point explored the substance connotation of excess and deficiency ZHENG with the metabonomic technology based on gas chromatography-mass spectrometry (GC-MS). The different substantial basis of two ZHENGs suggested that CHB patients could be categorized into two groups with diverse pathogenesis. The differential metabolites and disturbed pathways compared to not-obvious ZHENG characters patients (without ZHENG group/WZ) were selected in both of the two ZHENGs. The ROC analysis demonstrated that five metabolites had a greater potential to be the clinic biomarkers of EZ or DZ. And excess ZHENG revealed a higher level of immune function than deficiency ZHENG. We are eager to transform the concept of traditional excess and deficiency ZHENGs to modern therapeutic approaches, with the prospect to help to promote personalized medicine.

## 1. Introduction

As an important part of complementary and alternative medicine [1], traditional Chinese medicine(TCM) plays an important role in people's healthcare and is gaining in popularity [2] with its efficacy evidence increasing [3, 4]. It performs treatment based on ZHENG (translated as syndrome or pattern) Classification which is called “*bian zheng lun zhi*.” Owing to the manifestationin different syndromes, patients with the same disease would be treated differently by TCM practitioners [3], which is called “*tong bing yi zhi*” in Chinese. And one TCM therapy or formula should be specifically corresponded with a ZHENG, not a disease [5], and serious side effects could be caused by the abuse or misuse without any consideration of the ZHENG Classification [6, 7]. To demonstrate the importance of ZHENG classification, many studies have been conducted [8, 9]. For example, it was demonstrated in our previous experiment that if different ZHENGs of hepatitis-B-caused cirrhosis patients were treated by the same therapy, they would display various responses [10]. Furthermore, in a recent report, a biomedical therapy showed different effective rates for the rheumatoid arthritis patients with various TCM syndromes [11], which indicated that ZHENG was a subtype of the disease and would show some revelations for better individualized treatment in mainstream medicine. 

ZHENG depicts a whole pathological state of a patient by the profiles of symptoms, pulse feelings, and tongue appearance [12]. However, scientists cannot explain it with the mainstream medicine terminology. The introduction of system biology including genomics, proteomics, and metabonomics, facilitates the translation of TCM concepts into mainstream medicine based on biochemical, pathway or regulatory processes [13]. Lu et al. have built up the molecular networks of TCM cold and hot ZHENG in rheumatoid arthritis (RA) by network analysis [14]. Li et al. found the potential biomarkers of “Kidney-Yang deficiency syndrome” and the related disturbed pathway with the method of urine metabonomics [15]. 

Chronic hepatitis B (CHB) infection continues to be a global health problem [16]. As reported by World Health Organization (WHO) at 2012, an estimated 350 million people have been affected with CHB worldwide. About 600 000 people died every year due to the consequences of hepatitis B (http://www.who.int/mediacentre/factsheets/fs204/en/index.html). And the cure of CHB is never an easy task and there still remains further progress. TCM treatment guided by “*bian zheng lun zhi*” or combination with Westerntreatment can get a higher effective response rate [17]. Excess ZHENG (EZ) and deficiency ZHENG (DZ) is a double of basic ZHENGs of maladjustment body nature, reflecting the struggling state of human body and pathogenic factor. But the substantial basis or connotation of EZ and DZ is still unknown. The present paper designed a urine metabonomic study based on GC-MS to classify EZ and DZ patients with CHB patients and to map the substantial connotation of the two TCM ZHENGs by comparing EZ/DZ groups with the without ZHENG group (WZ) which was made up of the not-obvious ZHENG characters CHB patients. To our knowledge, this study is the first report of the classification of EZ and DZ based on CHB with urinary Metabonomics. 

## 2. Materials and Methods

### 2.1. Chemicals and Drugs

N,O-Bis(trimethylsilyl)trifluoroacetamide (BSTFA + TMCS 99 : 1) and urease were purchased from Sigma Corporation of American. Methoxyamine hydrochloride, heptane, methanol, ethanol, acetonitrile, acetone, chloroform and pyridine were analytical grade from China National Pharmaceutical Group Corporation in Shanghai of China. L-2-Chlorophenylalanine and heptadecanoic acid (also provided by Sigma Corporation of America) were used as an internal quality standard. The ultrapure water was obtained from a Milli-Q system (Millipore, USA). 

### 2.2. Subjects and Experiment Design

Three groups of CHB patients with EZ, DZ, and WZ and healthy volunteers group were enrolled in the study from Shuguang hospital of China. The numbers of cases were 30, 23, 30, and 20, respectively. All enrolled objects of the study were aged 18–65. The clinical study was approved by the local ethics committee and all of the recruited persons were given informed consent. And the study was performed in accordance with the principles contained in the Declaration of Helsinki. Diagnostic standard of HB patients was referred to “The guideline of prevention and treatment for chronic hepatitis B” [18]. TCM ZHENG classification was referred to as the viral hepatitis diagnostic standard described by the Internal Medicine Hepatopathy Committee of Chinese Traditional Medicine Association in December, 1991 [19]. All patients were diagnosed by junior Chinese medical physicians and then identified by three chief or deputy physicians; those who were consistently diagnosed as EZ or DZ by all of the physicians were enrolled in our study [20]. In addition, there is an exclusion criterion of CHB: (1) cases complicated with other hepatotropic virus hepatitis, (2) chronic severe hepatitis, (3) associated with serious primary disease of heart, kidney, lung, endocrine, blood, metabolic and gastrointestinal; or psychotic patients, (4) pregnant or lactating women. 

### 2.3. Sample Collection and Preparation

A complete physical examination was given; the health condition was recorded on a scale including the information obtained through four traditional examination methods: looking, listening and smelling, asking, and touching at the patient's entry into the study, and the urina sanguinis and vein blood were collected from all enrolled subjects. Urine and blood samples were stored at –80°C until GC-MS assay and the blood was utilized to detect the indicators of main medicine such as ALT and AST.

All these urine samples were thawed in ice water bath and vortex-mixed before analysis. Each 1 mL aliquot of standard mixture or urine sample was placed into a screw tube, 10 min centrifugation (12,000 rpm) was given, and aliquots of 150 *μ*L supernatant were transferred into another screw tube. After adding 70 *μ*L of urease (4 mg/mL) and vortex-mixing for 30 s, samples were conditioned at 37°C for 15 min to remove the urea. After the addition of 800 *μ*L of methanol and 10 *μ*L of myristic acid in methanol (1 mg/mL) and mixing for 1 min, the solution was centrifuged at 13,000 rpm for 10 min. Then a 200 *μ*L aliquot of supernatant was transferred into a GC vial and evaporated to dryness under N_2_ at 30°C. 50 *μ*L of methoxyamine in pyridine (15 mg/mL) was added to the GC vial, vortex-mixed for 1 min, and the methoximation reaction was carried out for 90 min rocking in a shaker at 30°C, then 50 *μ*L of BSTFA plus 1% TMCS was added to the samples for trimethylsilylation for another 1 h at 70°C. At last, 30 *μ*L of heptane containing external standard methyl myristate was added to the GC vial, and the solution was analyzed utilizing GC-MS after vortex for 30 s. 

### 2.4. Data Acquisition

One hundred and fifteen items of TCM symptoms and 67 items of biochemistry indicators of all objects were collected and recorded in the scales well designed before. and the list of 115 items of TCM symptoms and 67 items of biochemistry indicators could be found in the attachment with Supplementary Material available online at http://dx.doi.org/10.1155/2013/738245.

All GC-MS analyses were performed by a mass spectrometer 5975B (Agilent technologies, USA) coupled to an Agilent 6890 (Agilent technologies, USA) gas chromatography instrument. In the gas chromatographic system, a capillary column (Agilent J&W DB-5ms Ultra Inert 30 m × 0.25 mm, film thickness 0.25 *μ*m) was used. Helium carrier gas was used at a constant flow rate of 1.0 mL × min^–1^. One *μ*L of derivatized samples was injected into the GC/MS instrument, and splitless injection mode was used. A programmed column temperature was optimized to acquire a well separation, which was demonstrated in Table 1. The temperatures of the injection port, the interface, and source temperature were set at 280°C, 260°C, and 230°C, respectively. The measurements were made with electron impact ionization (70 eV) in the full scan mode (*m/z* 30–550). The solvent post time was set to 5 min. The GC-MS operating condition was the same as the previous experiment [10] except the column temperature program.

### 2.5. Data Analysis

Information of biochemical indicators and TCM symptoms was extracted from the scales and formed an excel matrix, then were analyzed in Smica-p11.5 Software (Umetrics, Umea, Sweden) for the analysis of principal component analysis (PCA), partial least squares discriminant analysis (PLS-DA), orthogonal projection to latent structures (OPLS) and spss 17.0 (SPSS, Chicago, IL, USA) for Mann-Whitney *U* test.

As to the profiles obtained from GC-MS, wispy shifts in retention time between fingerprints occur due to experimental variations and column aging. When the total ion current chromatograms (TICs) were obtained, peak-alignment or warping techniques are commonly applied to compensate for minor shifts in retention times. Thus, in the subsequently data processing, the same variable manifested synchronous information in every profile. So all the GC-MS raw files after being converted to CDF format via the software coming with Agilent MSD workstation, were subsequently processed by the XCMS toolbox (http://metlin.scripps.edu/download/) using XCMS's default settings with the following exceptions: xcmsSet (full width at half maximum: fwhm = 5; S/N cutoff value: snthresh = 10, max = 15), group (bw = 5). The resulting table (CSV file) was exported into Microsoft Excel (Microsoft Inc., USA), where normalization was performed prior to multivariate analyses. The resulting three-dimensional matrix involving peak index (RT-*m/z* pair), sample names (observations), and normalized peak area percent was introduced into Simca-P 11.5 Software (Umetrics, Umea, Sweden) for the analysis of PCA, PLS-DA, and OPLS. Differential variables with VIP values [21] exceeding 1.5 between two different groups could be generated from loadings plot. Subsequently, those variables were further analyzed by Mann-Whitney *U* test to confirm the changed metabolites by SPSS 17.0 (SPSS, Chicago, IL, USA) with the threshold of *P* value set at 0.05. Those variables, then, were identified by searching in NIST 2005 database and verified by standards. Kyoto Encyclopedia of Genes and Genomes (KEGG) (http://www.genome.ad.jp/kegg/) and Metabolites Biological Role (MBRole) (http://csbg.cnb.csic.es/mbrole) were based to select the related pathway. Many references were searched to give the biochemical interpretation of changed metabolites disturbed pathways of EZ and DZ in CHB.

## 3. Results

### 3.1. ZHENG Classification

#### 3.1.1. ZHENG Classification by Biochemical Indicators

Sixty-seven indicators of two groups of patients were analyzed by PCA, PLS-DA, and OPLS analyses attending to differentiate objects of EZ and DZ. Automatic modeling parameters indicated the poor explanation and predication of the models as shown in Table 2, meaning that the two ZHENGs could not be distinguished by profiles of biochemical indicators.

Table 3 showed us clinical information of two groups of CHB-affected patients based on western medical diagnostic approach. The commonly used indexes revealed no significant difference between the ZHENG groups by analysis of Mann-Whitney *U* test. It was illustrated that classification of EZ and DZ was irrelevant to these indexes.

#### 3.1.2. ZHENG Classification by Symptoms

**Figure 1:**
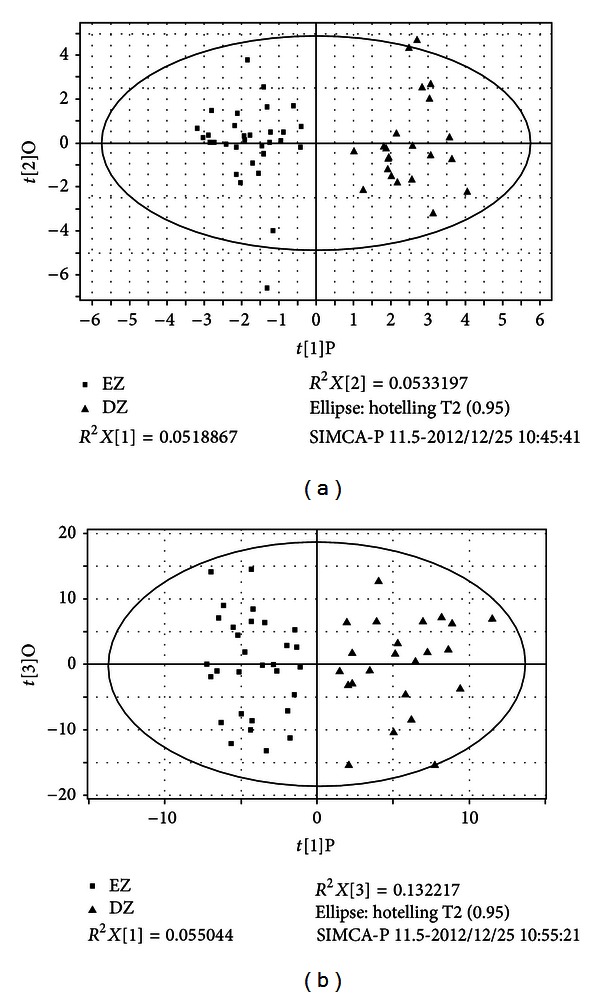
(a) Symptoms OPLS score plot of EZ and DZ. (b) Metabonomics OPLS score plot of EZ and DZ. EZ represents excess ZHENG patients group, DZ represents deficiency ZHENG patients group.

One hundred and fifteen TCM symptoms were analyzed by OPLS which could effectively extract variables responsible for the separation by removing variables unrelated to pathological status. Two groups could be absolutely separated as shown in the score plot (Figure 1(a)) with modeling (Model 1) information listed in Table 4.

#### 3.1.3. ZHENG Classification by Metabonomics

The matrix obtained by GC-MS based metabonomics could also classify the two ZHENG types in an OPLS score plot (Figure 1(b)). The model parameters are shown as Model 2 in Table 4. The plot showed that the two TCM types could not only be distinguished by TCM symptoms but also be classified by urine metabolic profiles, which was one of the evidences of material foundation of ZHENG. Subsequent analysis of differential metabolites and related pathways might explain the substance connotation of classification for these two ZHENGs. 

### 3.2. The Substance Connotation of DZ and EZ

#### 3.2.1. Metabolic Profiles of Chronic Hepatitis B Patients and Healthy Control

**Figure 2:**
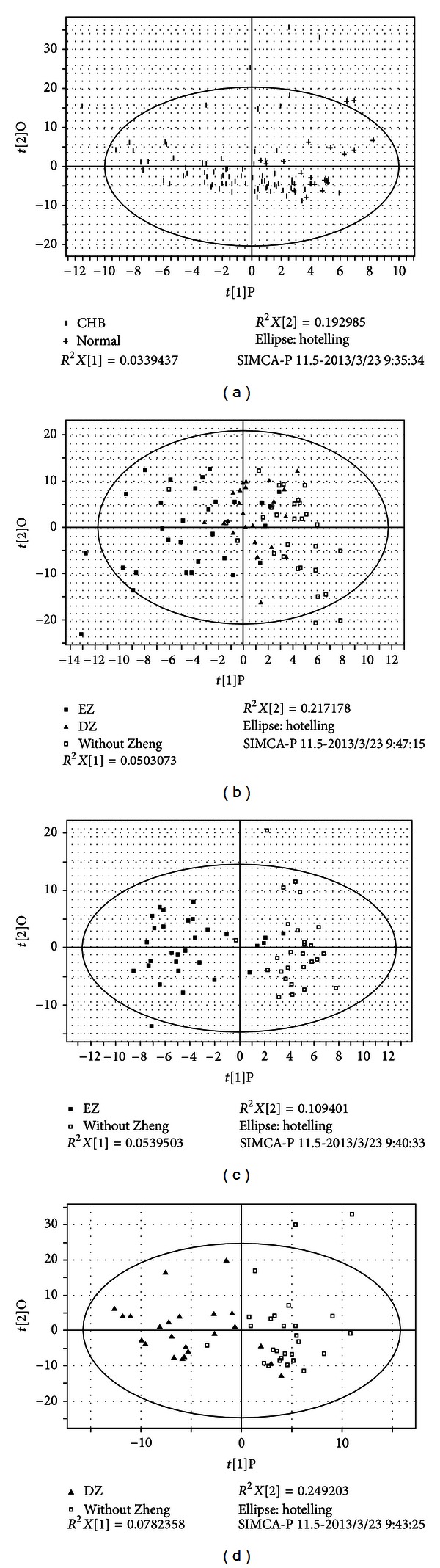
Metabonomics OPLS score plot, (a) CHB compared to normal group; (b) the comparison of EZ, DZ, and WZ; (c) EZ compared to WZ; (d) DZ compared to WZ. CHB means chronic hepatitis B group which includes excess ZHENG groupes, deficiency ZHENG group and WZ patients. EZ represents excess ZHENG patients groupes, DZ represents deficiency ZHENG patients group and WZ represents patients with not-obvious ZHENG characters.

The urine profiles of all the CHB patients and healthy volunteers (normal group) were analyzed by OPLS analysis. And Model 3 was calculated between CHB and normal group. The score plots were shown in Figure 2(a) and the modeling parameters were listed in Table 4. 

The three groups of CHB patients including EZ, DZ and WZ groups were also separated by OPLS analysis, which was depicted by the score plot (Figure 2(b)) and the modeling parameters were listed in Table 4 (Model 4). The results illustrated that different ZHENGs in CHB patients had their own substance basis or connotation. 

#### 3.2.2. Changed Metabolites Compared to WZ

**Figure 3:**
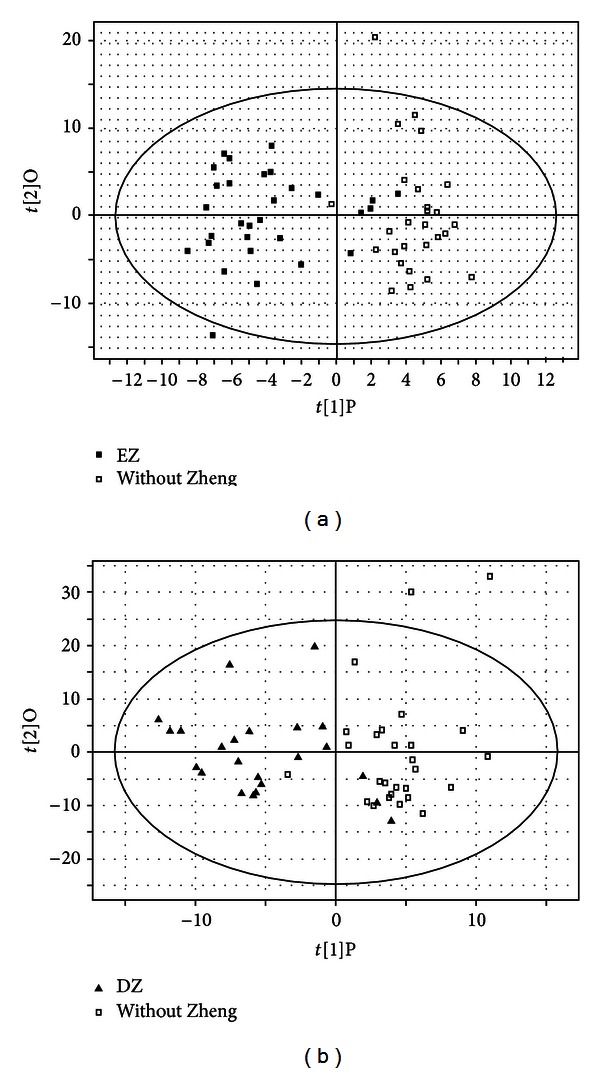
Metabonomics OPLS score plot, (a) EZ compared to WZ; (b) DZ compared to WZ.

**Figure 4:**
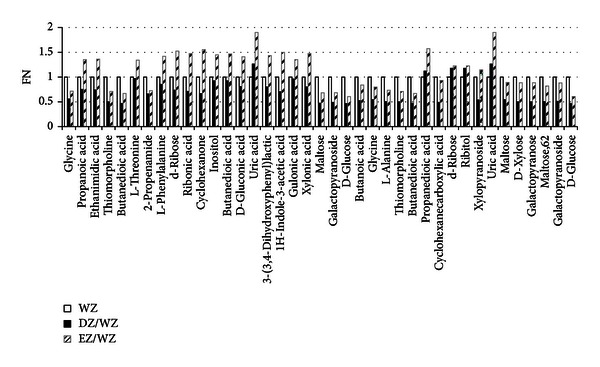
The differential metabolites level in EZ and DZ compared to WZ. All the differential metabolites were quantified by mean ranks obtained from Mann-Whitney *U* test firstly. Then the urinary metabolite relative levels of DZ and DZ were illustrated by fold change of mean ranks (FN) calculated by the differential metabolites of CHB with excess or deficiency ZHENG/the differential metabolites of CHB without ZHENG. The former 22 metabolites were calculated from EZ compared to WZ, and the later 17 metabolites were calculated from DZ compared to WZ.

The profiles from EZ (or DZ) CHB patients not only contained the information of different ZHENGs but were also involved in CHB disease. In order to explore the connotation of EZ and DZ, the disease factors should be eliminated. Therefore, data of EZ (or DZ) group was compared with data of WZ group which was made up of not-obvious ZHENG characters patients. The OPLS score plots were shown by Figure 3 and modeling parameters were listed in Table 4 (Model 5 and Model 6). The OPLS loading plots were made to screen out the specific variables contributing to the distinction between each ZHENG and WZ group. And the identified differential compounds and corresponding VIP values were listed in Table 5. All the differential compounds in the two ZHENGs were subsequently analyzed by Mann-Whitney *U* test compared to WZ group, respectively (*P* values were listed in Table 5) subsequently. The changing trend of differential metabolites was depicted by Figure 4.

#### 3.2.3. Sensitivity and Specificity of Potential Markers for TCM Syndrome Classification

**Figure 5:**
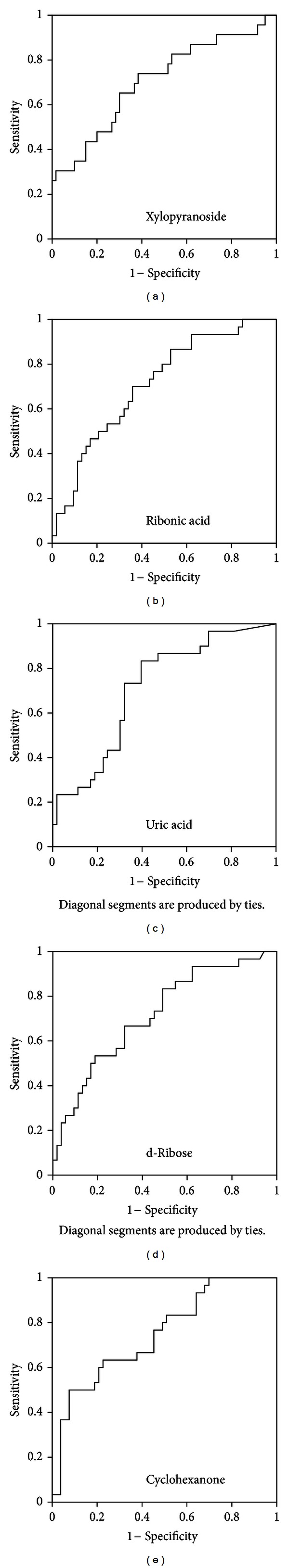
ROC curves for classification of two different TCM ZHENGs in CHB patients. The ROC curve of xylopyranoside (a) was generated by DZ group compared with non-DZ group, and the area under the curve is 0.705. ROC curves of ribonic acid (b), uric acid (c), d-ribose (d), and Cyclohexanone (e) were generated by EZ group compared with non-EZ group, and the AUCs were 0.711, 0.726, 0.732, and 0.744, respectively.

To determine the sensitivity and specificity of potential urine metabolic biomarkers of different TCM ZHENGs, ROC analysis was conducted. WZ group and DZ (/EZ) group were put together and defined as the non-EZ (/DZ) group, and so ROC analysis was carried out for discriminating EZ/DZ group with non-EZ/DZ group. The area under the ROC curves (AUC) for the differential metabolites was listed in Table 5. Among all the metabolites, the AUC values of xylopyranoside, ribonic acid, uric acid, d-ribose, and cyclohexanone fell into the range of 0.7–0.9. The ROC curves for classification of EZ/DZ group and non-EZ/DZ group were shown in Figure 5. It was suggested that the quantification of these five metabolites was more useful to classify excess and deficiency ZHENGs.

## 4. Discussion

In present work, it was the first time to stratify the EZ and DZ in CHB patients with the metabonomic technology. The results illustrate that the clinical biochemical indicators could not represent the characteristics of ZHENG. The clear separation between two groups by TCM symptoms and metabolic profiles illustrated that EZ and DZ had their substance fundaments. In order to eliminate the disease factors and explore the connotation of ZHENG, EZ (or DZ) group was compared with WZ group. Consequently, 22 and 17 differential metabolites from EZ and DZ were selected, respectively. The ROC curves of all the metabolites were conducted and five of them showed a higher sensitivity and specificity for the diagnosis of EZ or DZ (xylopyranoside for the diagnosis of DZ and ribonic acid, uric acid, d-Ribose and cyclohexanone for the diagnosis of EZ). It was suggested that these five metabolites were more potential to become the clinic biomarker of EZ or DZ. 

By searching in KEGG database and website of MBRole, the disturbed pathways in patients with either EZ or DZ were extracted based on the differential metabolites and were listed in Table 6. From the result, we could know that 6 pathways turned up imbalanced in both EZ, and DZ, 2 pathways were disturbed in EZ and 3 were disturbed in DZ with the same disease background. While the 6 common disturbed pathways revealed difference between EZ and DZ, either. For instance, glycine, d-ribose, maltose, galactopyranoside, and d-glucose are four related differential metabolites of ABC transporters, and they are all differential for EZ and DZ compared with WZ group. Functions of ABC transporters include the transport of toxic compounds [22]. while from bar diagram of Figure 4, we could get that the content of them was higher in EZ than in DZ which might be one of the manifestations of vital Qi deficiency in patients with DZ. EZ patients had a higher detoxified ability than DZ, which might correspond with the idea of “the vital Qi of EZ patient has not been deficient yet” in TCM theory. 

In addition, successful clearance of the virus as well as the formation of liver diseases was largely driven by a complex interaction between the virus and the host immune response in CHB patients [23], while the lower immune function in DZ than in EZ was reported in more than one research [24–26]. 

All above results suggest the ZHENG classification by symptoms profile has its fundaments. In other words, the pathomechanism and body state underlying different ZHENGs are discrepant, and the therapy and dosages should be tailored to each patient. The difference of immune function between EZ and DZ could interpret the TCM theory “treat excess by purgation and treat deficiency by tonification” to some extent. The present approach is transforming concepts of EZ and DZ to modern therapeutic approaches and will help to promote personalized therapy. 

Other limitations in this report are as follows. (1) The study was under a small sample size, and the lager population study should be further researched. (2) Because the first step is to differentiate excess and deficiency during the ZHENG diagnosis of CHB, only those two ZHENGs in CHB were discussed in this study. As two basic ZHENGs, however, either of them can be subdivided. For instance, liver-gallbladder dampness-heat ZHENG (one of EZ) has a high incidence rate of 12.1% [27] in CHB patients and its connotation awaits understanding.

## 5. Conclusion

The present paper firstly explored the classification and substance connotation of excess and deficiency ZHENG in CHB patients with metabonomic technology. We find that clinical biochemical indicators cannot represent the characteristics of the two ZHENGs because of poor classification. The TCM symptoms and urinary metabolic profiles can successfully distinguish the two ZHENGs. Twenty-two and seventeen differential metabolites were extracted from EZ and DZ, respectively, compared with WZ group. From the result of ROC curves, it was revealed that five differential metabolites had a greater potential to be the clinic biomarkers of EZ or DZ (xylopyranoside for the diagnosis of DZ and ribonic acid, uric acid, d-Ribose, and cyclohexanone for the diagnosis of EZ). And their related pathways were found, which showed the difference and intersection of their connotation. After further analysis, the immune function of EZ was demonstrated higher than that of DZ. The difference suggested that CHB patients could be categorized into two groups with diverse pathogenesis, which helps us transform the concept of traditional excess and deficiency ZHENGs to modern therapeutic approaches and prompts us to further individualized medicine. 

## Supplementary Material

The names of 115 items of TCM symptoms and 67 items of biochemistry indicators, which were collected from all the patients and volunteers recruited in this study

## Figures and Tables

**Table 1:** Temperature program of column incubator in GC/MS.

Rate (°C/min)	Temperature (°C)	Hold time (min)
	70	2
2.5	160	0
5	240	16

**Table 2:** Automatic modeling parameters for the classification of EZ and DZ.

Model	Amount of components	*R* ^2^ *X*	*R* ^2^ *Y*	*Q* ^2^ *Y*
PCA-X	1	0.111		0.0165
PLS-DA	1	0.0592	0.507	0.0723
OPLS	1	0.0592	0.508	0.0688

*R* ^2^ *X*_cum_ and *R* ^2^ *Y*_cum_ represent the cumulative sum of squares (SS) of all the *X*'s and *Y*'s explained by all extracted components.

*Q* ^2^ *Y*_cum_ is an estimate of how well the model predicts the *Y*'s.

**Table 3:** Clinical information from CHB-affected patients based on the WM diagnostic approach.

Indexes	Excess (*n* = 30)	Deficiency (*n* = 23)	*P*
Systolic pressure (mmHg)	114.90 ± 10.51	113.88 ± 8.23	0.378
Diastolic pressure (mmHg)	75.69 ± 7.86	76.6 ± 5.87	0.112
Age (years)	40.39 ± 14.58	39.5 ± 11.8	0.809
Gender (M/F)	31/7	25/9	0.277
ALT (IU/L)	61.26 ± 47.43	50.8 ± 55.4	0.378
AST (IU/L)	43.84 ± 19.6	45.96 ± 40.52	0.112
HBsAb (IU/mL)	2.20 ± 10.03	7.16 ± 28.96	0.107
TBIL (*µ*mol/L)	17.56 ± 7.57	17.43 ± 6.03	0.245
DBIL (*µ*mol/L)	6.1 ± 4.17	4.82 ± 1.82	0.817
IDBIL (*µ*mol/L)	11.45 ± 4.02	12.62 ± 4.92	0.062
GGT (IU/L)	49.55 ± 64.39	29.52 ± 14.68	0.811
ALP (IU/L)	94.71 ± 36.06	79.84 ± 20.81	0.489
TP (g/mL)	76.77 ± 7.99	79.34 ± 5.52	0.921
ALB (g/mL)	44.59 ± 4.4	45.99 ± 2.29	0.373

**Table 4:** Summary of the modeling information of OPLS analysis.

Name	No	*R* ^2^ *X*_cum_	*R* ^2^ *Y*_cum_	*Q* ^2^ *Y*_cum_
Model 1	1P + 1O	0.105	0.887	0.582
Model 2	1P + 3O	0.501	0.815	0.215
Model 3	1P + 2O	0.42	0.568	0.216
Model 4	1P + 4O	0.552	0.804	0.258
Model 5	1P + 2O	0.293	0.731	0.351
Model 6	1P + 2O	0.471	0.621	0.361

Model 1 was generated by EZ group compared with DZ group with TCM symptoms; Models 2–6 were generated by urine metabonomic data (Model 2: EZ group compared with DZ group; Model 3: CHB group compared with normal control; Model 4: comparison of EZ, DZ, and WZ; Model 5: EZ compared with WZ; Model 6: DZ compared with WZ.)

No represents the sum number of components. For instance, “1P + 1O” means one predictive component and two orthogonal components for establishing the OPLS model.

*R* ^2^ *X*_cum_ and *R* ^2^ *Y*_cum_ represent the cumulative sum of squares (SS) of all the *X*'s and *Y*'s explained by all extracted components.

*Q* ^2^ *Y*_cum_ is an estimate of how well the model predicts the *Y*'s.

**Table 5:** Differential metabolites in EZ and DZ, respectively, compared to WZ group.

Group	Changed metabolites	VIP	*P*_(M-W)_	FN_DZ/WZ_	FN_EZ/WZ_	AUC_ROC_
EZ	Glycine	2.10	0.025	0.56	0.72	0.632
EZ	Propanoic acid	1.74	0.043	0.75	1.35	0.678
EZ	Ethanimidic acid	1.88	0.039	0.74	1.36	0.671
EZ	Thiomorpholine	2.08	0.021	0.50	0.71	0.588
EZ	Butanedioic acid	2.24	0.007	0.47	0.67	0.601
EZ	L-Threonine	1.64	0.049	0.97	1.34	0.618
EZ	2-Propenamide	1.66	0.03	0.66	0.72	0.672
EZ	L-Phenylalanine	2.11	0.019	0.85	1.42	0.655
EZ	D-Ribose	1.86	0.005	0.74	1.52	0.732
EZ	Ribonic acid	1.70	0.01	0.71	1.47	0.711
EZ	Cyclohexanone	2.03	0.003	0.66	1.55	0.744
EZ	Inositol	1.71	0.014	0.92	1.45	0.649
EZ	Butanedioic acid	1.71	0.011	0.92	1.47	0.65
EZ	D-Gluconic acid	1.93	0.012	0.81	1.41	0.661
EZ	Uric acid	2.45	0.000	1.26	1.90	0.726
EZ	3-(3,4-Dihydroxyphenyl)lactic acid	2.03	0.016	0.80	1.43	0.66
EZ	1H-Indole-3-acetic acid	1.96	0.007	0.70	1.50	0.695
EZ	Gulonic acid	1.62	0.042	0.96	1.35	0.619
EZ	Xylonic acid	2.03	0.01	0.80	1.47	0.697
EZ	Maltose	2.08	0.01	0.47	0.68	0.577
EZ	Galactopyranoside	2.07	0.011	0.49	0.68	0.584
EZ	D-Glucose	2.5	0.001	0.46	0.61	0.617
DZ	Butanoic acid	1.55	0.02	0.52	0.84	0.655
DZ	Glycine	1.53	0.034	0.54	0.80	0.604
DZ	L-Alanine	1.91	0.01	0.50	0.73	0.639
DZ	Thiomorpholine	2.04	0.009	0.50	0.71	0.632
DZ	Butanedioic acid	1.92	0.003	0.47	0.67	0.641
DZ	Propanedioic acid	1.22	0.016	1.12	1.57	0.589
DZ	Cyclohexanecarboxylic acid	1.62	0.007	0.49	0.93	0.695
DZ	D-Ribose	1.51	0.006	1.18	1.22	0.675
DZ	Ribitol	1.42	0.006	1.18	1.22	0.675
DZ	Xylopyranoside	1.23	0.029	0.54	1.15	0.705
DZ	Uric acid	2.39	0.001	1.26	1.90	0.591
DZ	Maltose	1.41	0.034	0.54	0.89	0.646
DZ	D-Xylose	1.76	0.009	0.50	0.88	0.678
DZ	Galactopyranose	1.78	0.009	0.50	0.88	0.678
DZ	Maltose,62	1.71	0.011	0.50	0.82	0.654
DZ	Galactopyranoside	1.69	0.013	0.51	0.88	0.665
DZ	D-Glucose	2.04	0.003	0.46	0.61	0.658

EZ represents excess ZHENG patients group, DZ represents deficiency ZHENG patients group.

VIP means the variable importance in the project.

*P*_(M-W)_ value was obtained from Mann-Whitney test (ZHENGs compared to WZ group).

FN_DZ/WZ_ or FN_DZ/WZ_ is fold change of mean ranks calculated by the differential metabolites of CHB with excess or deficiency ZHENG/the differential metabolites of CHB without ZHENG.

AUC_ROC_ means the area under the ROC curve.

**Table 6:** Disturbed pathways of EZ and DZ compared to WZ group.

Pathway	*P*-val EZ versus WZ	*P*-val DZ versus WZ	Class	Annotation
ABC transporters	<0.0001	<0.0001	Environmental information processing	Two ZHENGs in common
Pentose phosphate pathway	<0.0001	<0.0001	Carbohydrate metabolism	Two ZHENGs in common
Aminoacyl-tRNA biosynthesis	0.003	0.006	Carbohydrate metabolism	Two ZHENGs in common
Galactose metabolism	0.012	0.009	Carbohydrate metabolism	Two ZHENGs in common
Starch and sucrose metabolism	0.018	0.015	Carbohydrate metabolism	Two ZHENGs in common
Pentose and glucuronate interconversions	0.020	0.029	Genetic information processing	Two ZHENGs in common
Ascorbate and aldarate metabolism	<0.0001	>0.05	Carbohydrate metabolism	Specific for EZ
Glycine, serine, and threonine metabolism	0.017	>0.05	Amino acid metabolism	Specific for EZ
Alanine, aspartate, and glutamate metabolism	>0.05	0.003	Amino acid metabolism	Specific for DZ
Amino sugar and nucleotide sugar metabolism	>0.05	0.003	Carbohydrate metabolism	Specific for DZ
Glycolysis/gluconeogenesis	>0.05	0.005	Carbohydrate metabolism	Specific for DZ
Purine metabolism	>0.05	0.042	Nucleotide metabolism	Specific for DZ

*P*-val: the statistical significance of each pathway obtained from analysis of MBRole.

Class: categorization of each pathway obtained from KEGG.
