# Supplementary material for: Differences of Excess and Deficiency Zheng in Patients with Chronic Hepatitis B by Urinary Metabonomics
Source: Evid Based Complement Alternat Med. 2013 Apr 24;2013:738245. doi: 10.1155/2013/738245 (PMC3655602; doi:10.1155/2013/738245)
Supplement: Supplementary file 1 — The names of 115 items of TCM symptoms and 67 items of biochemistry indicators, which were collected from all the patients and volunteers recruited in this study [file 738245.f1.pdf]

1. 115 items of TCM symptoms

No	TCM symptoms	Abbreviation
1	Resting heart rate	Resting_heart_rate
2	Systolic blood pressure	Ssy
3	Diastolic blood pressure	Szy
4	Headache	A0101
5	Dizziness	A0102
6	Dizziness heavy	A0103
7	Giddiness	A0104
8	Eyelids heavy	A0105
9	Depending on the material fatigue	A0106
10	Red eyes	A0107
11	Head dry	A0108
12	Tinnitus	A0109
13	Epistaxis	A0110
14	Dry nose	A0111
15	Dark lip Violet	A0112
16	Bitter taste in the mouth	A0113
17	Pale mouth	A0114
18	Tired mouth	A0115
19	Thirst	A0116
20	Bad breath	A0117
21	Mouth sores	A0118
22	Bleeding gums	A0119
23	Sore gums	A0120
24	Loose teeth	A0121
25	Dry throat	A0122
26	Sore throat	A0123
27	Taixi	A0124
28	Yawn	A0125
29	Eructation	A0126
30	Spontaneous	A0127
31	Night sweat	A0128
32	Palpitations	A0129
33	Chest tightness	A0130
34	Sleepiness	A0131
35	Insomnia	A0132
36	More than a dream	A0133
37	Forgetful	A0134
38	Bellyache	A0135
39	Bloating	A0136
40	Epigastric pain	A0137
41	Epigastric pain	A0138

**42**	**Epigastric tingling**	**A0139**
**43**	**Tunsuan**	**A0140**
**44**	**Abdominal nausea**	**A0141**
**45**	**Anorexia**	**A0142**
**46**	**Nausea and vomiting**	**A0143**
**47**	**Hiccups**	**A0144**
**48**	**Lassitude**	**A0145**
**49**	**Flank pain**	**A0146**
**50**	**Flank pain**	**A0147**
**51**	**Flank tingling**	**A0148**
**52**	**Irritability**	**A0149**
**53**	**Lumbago**	**A0150**
**54**	**Waist cold**	**A0151**
**55**	**Backache**	**A0152**
**56**	**Soft knee**	**A0153**
**57**	**Five upset hot**	**A0154**
**58**	**Afternoon hot flashes**	**A0155**
**59**	**Bowel**	**A0156**
**60**	**Loose stools**	**A0157**
**61**	**Constipation**	**A0158**
**62**	**Stool foul smell**	**A0159**
**63**	**Tenesmus**	**A0160**
**64**	**Nocturia Kiyonaga**	**A0161**
**65**	**Yellow urine**	**A0162**
**66**	**Who heads yellow**	**A0163**
**67**	**Pale complexion**	**A0164**
**68**	**Looking dull**	**A0165**
**69**	**Pale white**	**A0166**
**70**	**Facial redness**	**A0167**
**71**	**Angioma**	**A0168**
**72**	**Liver palms**	**A0169**
**73**	**Itchy skin**	**A0170**
**74**	**Skin a mistake**	**A0171**
**75**	**Skin purpura**	**A0172**
**76**	**Numbness**	**A0173**
**77**	**Limb heaviness**	**A0174**
**78**	**Joint pain**	**A0175**
**79**	**Men's wet dream**	**A0176**
**80**	**Menstrual flow**	**A0177**
**81**	**Menstruation**	**A0178**
**82**	**Vaginal discharge volume**	**A0179**
**83**	**Vaginal discharge gas smell**	**A0180**
**84**	**Tongue color**	**t_color**

**85**	**Tongue color parts**	**t_color_position**
**86**	**Noted in the tongue color**	**t_color_notes**
**87**	**Ups and downs**	**t_shape_1**
**88**	**Laonen**	**t_shape_2**
**89**	**Fat, thin**	**t_shape_3**
**90**	**Indented**	**t_shape_4**
**91**	**Prick**	**t_shape_5**
**92**	**Crack**	**t_shape_6**
**93**	**Ecchymosis**	**t_shape_7**
**94**	**Bohou**	**t_c_qlty_1**
**95**	**Run less Tianjin dry rough**	**t_c_qlty_2**
**96**	**Tired of rot**	**t_c_qlty_3**
**97**	**Less moss no moss**	**t_c_qlty_4**
**98**	**Exfoliative**	**t_c_qlty_5**
**99**	**Moss quality parts**	**t_c_q_position**
**100**	**An moss qualitative marked**	**t_c_q_notes**
**101**	**Moss color**	**t_coat**
**102**	**Moss color parts**	**t_coat_position**
**103**	**Noted in the moss color**	**t_coat_notes**
**104**	**Sublingual vein**	**t_vein**
**105**	**Ups and downs**	**pulse_1**
**106**	**fast and slow**	**pulse_2**
**107**	**deficiency and excess**	**pulse_3**
**108**	**Length of pulse**	**pulse_4**
**109**	**string pulse**	**pulse_5**
**110**	**thin pulse**	**pulse_6**
**111**	**Slippery pulse**	**pulse_7**
**112**	**Mild pulse**	**pulse_8**
**113**	**slow pulse**	**pulse_9**
**114**	**Astringent pulse**	**pulse_10**
**115**	**Weak pulse**	**pulse_11**

2. 67 items of biochemistry indicators

**Number**	**indicators**	**Number**	**indicators**
**1**	**TBIL**	**35**	**Uric acid**
**2**	**DBIL**	**36**	**Glucose**
**3**	**IDBIL**	**37**	**TC**
**4**	**ALT**	**38**	**TG**
**5**	**AST**	**39**	**HDL-C**
**6**	**GGT**	**40**	**LDL-C**
**7**	**ALP**	**41**	**APOA-1**
**8**	**TP**	**42**	**Albumin**
**9**	**ALB**	**43**	**α1 globulin**

**10**	**Pre-Alb**	**44**	**α2 globulin**
**11**	**Bile acid**	**45**	**β-globin**
**12**	**PT**	**46**	**γ-globulin**
**13**	**TT**	**47**	**Basophil**
**14**	**APTT**	**48**	**Basophil%**
**15**	**HBsAg**	**49**	**Eosinophils**
**16**	**HBsAb**	**50**	**Eosinophils%**
**17**	**HBeAg**	**51**	**Hemoglobin**
**18**	**HBeAb**	**52**	**Lymphocytes**
**19**	**HBcAb**	**53**	**Lymphocytes%**
**20**	**HbcAb-IgM**	**54**	**Mean hemoglobin**
**21**	**Pre-S1 antigen**	**55**	**The average hemoglobin concentration**
**22**	**Pre-S1 antibodies**	**56**	**Mean corpuscular volume**
**23**	**HBV-DNA**	**57**	**Monocytes**
**24**	**Protein**	**58**	**% Of monocytes**
**25**	**Hand-protein**	**59**	**Mean platelet volume**
**26**	**Proportion**	**60**	**Neutrophil**
**27**	**CD3**	**61**	**Neutrophils%**
**28**	**CD4 +**	**62**	**Platelet hematocrit**
**29**	**CD8 +**	**63**	**Platelet distribution width**
**30**	**IgA**	**64**	**Blood platelet**
**31**	**IgM**	**65**	**Erythrocyte**
**32**	**IgG**	**66**	**Red cell distribution width**
**33**	**BUN**	**67**	**Leukocyte**
**34**	**Cr**		
